# Genotypic Categorization of Loeys-Dietz Syndrome Based on 24 Novel Families and Literature Data

**DOI:** 10.3390/genes10100764

**Published:** 2019-09-28

**Authors:** Letizia Camerota, Marco Ritelli, Anita Wischmeijer, Silvia Majore, Valeria Cinquina, Paola Fortugno, Rosanna Monetta, Laura Gigante, Marfan Syndrome Study Group Tor Vergata University Hospital, Federica Carla Sangiuolo, Giuseppe Novelli, Marina Colombi, Francesco Brancati

**Affiliations:** 1 Human Genetics Institute, Department of Life, Health, and Environmental Sciences, University of L’Aquila, 67100 L’Aquila, Italy; letizia.camerota@guest.univaq.it (L.C.);; 2 Division of Biology and Genetics, Department of Molecular and Translational Medicine, University of Brescia, 25123 Brescia, Italy; marco.ritelli@unibs.it (M.R.); valeria.cinquina1@unibs.it (V.C.); 3 Clinical Genetics Unit, Department of Pediatrics, Regional Hospital of Bolzano, 39100 Bolzano, Italy; titiaanita.wischmeijer@sabes.it; 4 Medical Genetics Laboratory, Department of Molecular Medicine, Sapienza University, 00185 Rome, Italy; smajore@scamilloforlanini.rm.it; 5 San Camillo-Forlanini Hospital, 00152 Rome, Italy; 6 Laboratory of Molecular and Cell Biology, Istituto Dermopatico dell’Immacolata, IDI-IRCCS, 00167 Rome, Italy; 7 Department of Biomedicine and Prevention, Tor Vergata University, 00133 Rome, Italy; laura.gigante84@gmail.com (L.G.); sangiuolo@med.uniroma2.it (F.C.S.); novelli@med.uniroma2.it (G.N.); 8 Medical Genetics Unit, Policlinico Tor Vergata University Hospital, 00133 Rome, Italy; 9 IRCCS Neuromed Institute, 86077 Pozzilli, Italy

**Keywords:** hereditary connective tissue disorders, Loeys-Dietz syndrome, Ehlers-Danlos syndrome, arterial aneurysms, *TGFBR1*, *TGFBR2*, *SMAD2*, *SMAD3*, *TGFB2*, *TGFB3*

## Abstract

Loeys-Dietz syndrome (LDS) is a connective tissue disorder first described in 2005 featuring aortic/arterial aneurysms, dissections, and tortuosity associated with craniofacial, osteoarticular, musculoskeletal, and cutaneous manifestations. Heterozygous mutations in 6 genes (*TGFBR1/2, TGFB2/3, SMAD2/3*), encoding components of the TGF-β pathway, cause LDS. Such genetic heterogeneity mirrors broad phenotypic variability with significant differences, especially in terms of the age of onset, penetrance, and severity of life-threatening vascular manifestations and multiorgan involvement, indicating the need to obtain genotype-to-phenotype correlations for personalized management and counseling. Herein, we report on a cohort of 34 LDS patients from 24 families all receiving a molecular diagnosis. Fifteen variants were novel, affecting the *TGFBR1* (6), *TGFBR2* (6), *SMAD3* (2), and *TGFB2* (1) genes. Clinical features were scored for each distinct gene and matched with literature data to strengthen genotype-phenotype correlations such as more severe vascular manifestations in *TGFBR1/2*-related LDS. Additional features included spontaneous pneumothorax in *SMAD3*-related LDS and cervical spine instability in *TGFB2*-related LDS. Our study broadens the clinical and molecular spectrum of LDS and indicates that a phenotypic continuum emerges as more patients are described, although genotype-phenotype correlations may still contribute to clinical management.

## 1. Introduction

Loeys–Dietz syndrome (LDS) is a rare hereditary connective tissue disorder (HCTD) with an autosomal dominant inheritance characterized by a widespread systemic involvement. The disorder was first described in 2005 and in its most typical presentation it features aortic/arterial aneurysms and/or dissections, as well as arterial tortuosity in association with variable craniofacial, osteoarticular, musculoskeletal, and cutaneous manifestations [1,2]. LDS is caused by pathogenic variants in transforming growth factor β (TGF-β) signaling pathway-related genes, i.e., *TGFBR1*, *TGFBR2*, *SMAD2*, *SMAD3*, *TGFB2,* and *TGFB3*, which alter the physiological development and function of the extracellular matrix, leading to cardiovascular and multisystem abnormalities [3]. Mutations in the genes encoding the TGF-β receptor subunits (*TGFBR1* and *TGFBR2*) were firstly identified and an initial clinical classification of LDS type I and type II was proposed based on the presence of typical craniofacial features in LDS type I [2]. Over the years, the discovery of novel disease-causative genes, eased by the advent of next-generation sequencing techniques, paved the way for novel genotype-phenotype correlations. For example, the identification of a subset of LDS patients with pathogenic variants in *SMAD3* displaying high frequency of osteoarthritis, prompted some authors to define the resulting phenotype as “aneurysms-osteoarthritis syndrome” or LDS type III [4]. McCarrick and coworkers argued that this phenotype fits within the LDS spectrum based on the analysis of large cohorts of mutated patients [5]. In particular, the authors concluded that, in the absence of formal diagnostic criteria for LDS, a pathogenic variant in any of the six LDS disease-causative genes in combination with the presence of arterial aneurysm or dissection or a positive family history should be considered sufficient for the diagnosis of LDS [5]. This stimulated the study of genotype-phenotype correlations, which are of paramount value for correct management of patients and to plan appropriate prevention programs in clinical practice. Even if wide inter- and intrafamilial variability in the distribution and severity of clinical features is observed, a more accurate gene-based categorization of LDS may have a great clinical impact in early diagnosis and management and guide treatment strategies for patients and family members with this life-threatening condition. Of note, the diagnosis of patients with LDS prompting molecular testing is not always straightforward, since the spectrum of clinical manifestations is broad and often overlaps other HCTDs, including Marfan syndrome (MFS), Shprintzen-Goldberg syndrome, some types of cutis laxa, Ehlers-Danlos syndromes (EDS) (particularly the vascular type), arterial tortuosity syndrome, congenital contractual arachnodactyly, and biglycan (*BGN*)-associated aortic aneurysm syndrome. Additionally, in some cases further genetic testing may be indicated for the differential diagnosis with other hereditary thoracic aortic disorders caused by pathogenic variants in distinct genes including *NOTCH1*, *ACTA2*, *MYH11*, *MYLK*, *PRKG1*, *MAT2A*, *FOXE3*, *MFAP5*, and *LOX* [6]. Herein, we describe 34 patients from 24 families with LDS in which we identified the genetic defect in one of the known genes; 15 variants were novel, expanding the mutational repertoire. We further strengthened genotype-phenotype correlations by fitting our data with available studies in literature and updated the gene-to-phenotype categorization of LDS.

## 2. Patients and Methods

### 2.1. Patients

Twenty-four index patients and 10 relatives with LDS, mostly of Italian origin but two from Philippines and Sri Lanka, were evaluated from 2010 to 2018 in specialized outpatient clinics for the diagnosis of HCTDs, namely: (*i*) the Ehlers-Danlos Syndrome and Inherited Connective Tissue Disorders Clinic at the University Hospital Spedali Civili of Brescia, (*ii*) the Medical Genetics Unit of the Sant’Orsola-Malpighi Hospital of Bologna, (*iii*) the Clinical Genetics Unit of the Regional Hospital of Bolzano, (*iv*) the Centre of Expertise for Marfan syndrome and Marfan-related disorders at Policlinico Tor Vergata University Hospital of Rome and (*v*) the Medical Genetics Unit, Department of Life, Health, and Environmental Sciences of the University of L’Aquila. 

### 2.2. Molecular Investigations

Molecular analyses were performed in the laboratory of genetic testing at the Division of Biology and Genetics, Department of Molecular and Translational Medicine, University of Brescia. Mutational screening was achieved on genomic DNA purified from peripheral blood leukocytes of affected and unaffected family members by standard procedures. All exons and their intron-flanking regions of *TGFBR1* (NM_004612.4, NP_004603.1), *TGFBR2* (NM_003242.5, NP_0033233.4), *SMAD*3 (NM_003242.5, NP_005893.1), and *TGFB2* (NM_001135599.3, NP_001129071.1) were PCR amplified by using optimized genomic primers (Supplementary Table S1, primers were designed by using the Primer Express software v. 3.0.1 (Thermo Fisher Scientific, South San Francisco, CA, USA) and purchased by Metabion International AG, Planneg, Germany), which were analyzed for the absence of known variants using the GnomAD database [7]. After enzymatic cleanup of the PCR products, all fragments were sequenced in both orientations using the Big Dye Terminator Cycle Sequencing kit protocol (Thermo Fisher Scientific, South San Francisco, CA, USA) followed by capillary electrophoresis on the ABI3130XL Genetic analyzer. The sequences were analyzed with the Sequencher 5.0 software (Gene Codes Corporation, Ann Arbor, MI, USA) and variants were annotated according to the Human Genome Variation Society (HGVS) nomenclature with the Alamut Visual software version 2.11 (Interactive Biosoftware, Rouen, France), which was also used for splice site prediction, since it includes four different prediction algorithms: SpliceSiteFinder-like, MaxEntScan, NNSplice, and GeneSplicer. When available, fresh blood samples were collected in patients carrying variants affecting canonical splice sites. In order to verify the effect on splicing, RT-PCR was carried out on total RNA extracted from patients’ whole blood stabilized in Paxgene tubes following the manufacturer’s protocol by using the Paxgene Blood RNA Extraction Kit (PreAnalytiX, Qiagen, Hilden, Germany). In particular, amplification of cDNA covering exons 2–4 of *TGFBR2* (TGFBRex2-forw: 5′-GTGGCTGTATGGTAAGAGA-3′ and TGFBR2ex4-rev: 5′-CCAGGTTGAACTCAGCTTCTG-3′) and exons 6-8 of *SMAD3* (SMAD3ex6-forw: 5′-CCTAGGGCTGCTCTCCAATG-3′ and SMAD3ex8-rev: 5′-GTGCACATTCGGGTCAACTG-3′) was performed, respectively, and followed by Sanger sequencing of the RT-PCR products. All identified variants were submitted to the Leiden Open Variation Database (LOVD) [8].

### 2.3. Genotype-Phenotype Analysis and Literature Review

The medical records of 34 patients with a molecularly proven diagnosis of LDS were accessed and their phenotypes were defined using the Human Phenotype Ontology (HPO) terms by a careful review of clinical notes. Phenotypic categories were created based on the number of observations in the patient’s cohort and their relationship to LDS. In reporting the frequency of clinical features of our case series, the used denominator refers to the number of subjects for which information on a specific feature was available (Table 1). We reviewed the medical literature on the clinical manifestations of patients with a confirmed genetic diagnosis of LDS, i.e., patients from large cohorts harboring mutations in one of the six known disease-causative genes. The primary search was completed in the PubMed database and limited to articles in the English language literature without restrictions on the date of publication. Keywords used were “Loeys-Dietz syndrome” AND “genotype”, “Loeys-Dietz syndrome” AND “phenotype” as well as “Loeys-Dietz syndrome” AND “review.” A secondary search was performed to identify pertinent articles cited in those selected in the primary search. We tried to identify all papers for which genotype-phenotype correlation data were available, focusing on reviews and large cohort studies. 

### 2.4. Ethical Compliance 

This study was approved by the relevant Ethical Authorities of the: Policlinico Tor Vergata University Hospital (Progetto di Ricerca PGR00229, R.S. 204/16); University of L’Aquila, Medical Genetics Section, Department of Life, Health, and Environmental Sciences (PGR00229, prot. 20251); and local Ethical Committees. This study was performed in agreement with the principles of the 1964 Helsinki Declaration. All subjects (or their legally authorized representative) enrolled into the genetic study provided written informed consent.

## 3. Results

### 3.1. Demographic Data and Genotype-Phenotype Analysis of LDS Patient’s Cohort

**Figure 1:**
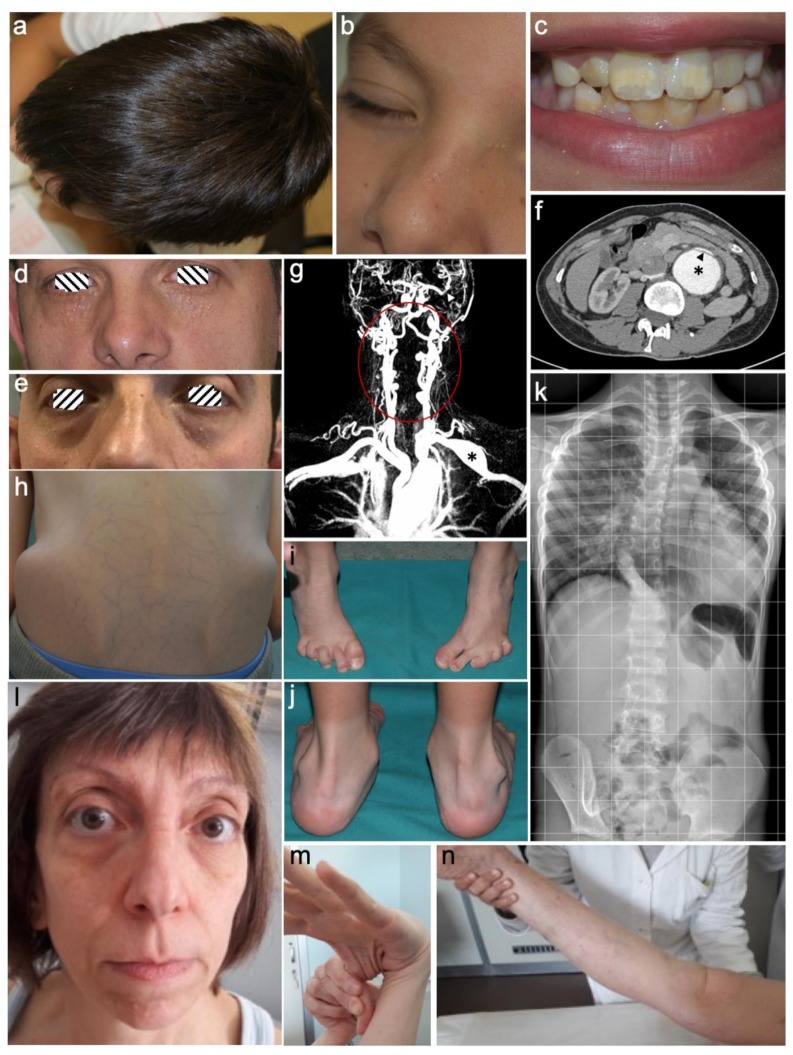
Clinical and instrumental findings observed in our Loeys-Dietz syndrome (LDS) patients. (**a**–**c**) Proband of family 1 (p.Asp400Gly,*TGFBR1*), aged 7 years manifesting dolichocephaly (**a**), milia (**b**) and enamel defect of permanent dentition. (**d**,**e**) Family 4 (p.Gly271Asp, *TGFBR1*), proband (**d**) and his brother (**e**) outlining intrafamilial variability of palpebral fissures, horizontal in the proband (**d**) and downslanted in his brother (**e**) in the presence of severe milia in both. (**f**) CT-angiography of the abdomen in the proband of family 7 (p.Gly353Arg, *TGFBR1*), aged 23 years, showing aneurysm of the abdominal aorta (asterisk) with intimal blister flap (arrow head). (**g**) Proband of family 15 (p.Asp522Asn, *TGFBR2*), aged 36 years: CT-angiography of head and neck showing fusiform aneurysm of the left subclavian artery (asterisk), middle cerebral artery stenosis (arrow heads), diffuse tortuosity of vertebral arteries (area within the circle). (**h**–**k**) Proband of family 16 (p.Asp446Asn, *TGFBR2*), aged 7 years, displays asymmetry of iliac crest, thin and translucent skin with highly visible subcutaneous venous reticulum (**h**); Limited function of the feet after corrective surgery for bilateral clubfoot (**i**); hind foot deformity with bilateral pronated valgus pes planus (**j**); X-ray frontal view performed at 6 years shows the displaced heart in the left thorax due to severe pectus excavatum and scoliosis with lumbar left rotation and asymmetric iliac wings (**k**). (**l**–**n**), Proband of family 24 (p.Phe160Leufs*14, *TGFB2*), aged 48 years featuring facial asymmetry and dysmorphism (highly arched eyebrows, hypertelorism, bilateral exophthalmos, bifid nasal tip, long philtrum with thin upper lip, micrognathia) in addition to premature aging appearance (**l**); Joint laxity of the thumb (**m**) and the knee (**n**); Note the thin and translucent skin with visible subcutaneous veins.

We report the clinical and genetic features of 34 individuals from 24 families. The female-to-male ratio of affected probands was 1/1. The mean age at diagnosis of the 24 unrelated probands was 26 years (range 1–51 years). Thirteen of them had a positive family history, while 11 were sporadic. Ten individuals were diagnosed upon familial segregation. The detailed clinical and molecular features of each affected individual are summarized in Supplementary Table S2. The overall frequency of selected clinical features observed in our cohort, categorized for each distinct gene as compared to in the literature, is shown in Table 1. An overview of characteristic features observed in the patients of our cohort is presented in Figure 1.

### 3.2. Molecular Findings

In this study, 24 different variants in four distinct LDS-related genes were identified in the proband of each family by direct sequencing (Table 2). All variants were deposited in LOVD. The majority of variants affected the *TGFBR1* (9/24) and *TGFBR2* (10/24) genes, four families showed variants in *SMAD3*, and 1 patient harbored a *TGFB2* variant. No variants were identified in *SMAD2* and *TGFB3*. Fifteen variants affecting *TGFBR1*, *TGFBR2*, *SMAD3*, and *TGFB2* were novel, while nine in *TGFBR1*, *TGFBR2*, *SMAD3* were previously reported either in the literature or in the ClinVar database (Supplementary Table S2 and Table 2).

Among novel variants, 10 missense substitutions and one in-frame deletion of two highly conserved amino acid residues p.(Lys232_Ile233del) were evaluated for their putative pathogenicity through different *in silico* prediction algorithms, all predicted them as being high impacting variants. Given that they are: (i) located in critical and well-established functional domains without benign variation; (ii) absent in publicly available population databases, (iii) predicted as deleterious my multiple lines of computational evidence and based on the matching of the clinical phenotypes, all these variants were classified as pathogenic (class 5) according to the American College of Medical Genetics and Genomics (ACMG) guidelines [9]. 

Likewise, the novel c.480del variant in *TGFB2* was classified as pathogenic (class 5), since it leads to frameshift and formation of a premature termination codon p.(Phe160Leufs*14) that likely activates nonsense-mediated mRNA decay (NMD). 

For two out of three variants affecting splice sites, we obtained fresh blood samples and demonstrated an effect on the splicing process, corroborating their pathogenicity. In particular, the c.263+6C>T variant in *TGFBR2* (rs758501054, Minor allele frequency in ExAC, MAF = 0.000008) was classified as likely pathogenic (class 4) according to the ACMG, since it creates a new splice donor site 6 bases downstream of the wild-type donor with retention of 4 nucleotides of intron 3, formation of a stop codon (p.Arg114*) and activation of NMD. The c.1009+1G>A variant in *SMAD3* was classified as pathogenic (class 5), since it abolishes the canonical splice donor site of exon 7 causing in-frame exon skipping (p.Arg292_Gly337del). Lastly, although RNA was not available to investigate its effect, the c.862_871+8del pathogenic variant (class 5) variant in *SMAD3,* thought formally a frameshift variant, likely leads to abnormal splicing as well (i.e., in-frame exon 6 skipping), as the canonical splice donor site is lost.

## 4. Discussion

Since the relatively recent description of LDS in 2005, six disease-causative genes have been identified, namely *TGFBR1* (MIM #190181) [1], *TGFBR2* (MIM#190182) [1], *SMAD2* (MIM #601366) [10], *SMAD3* (MIM #603109) [4], *TGFB2* (MIM #190220) [11], and *TGFB3* (MIM#190230) [12]. This advised the subdivision of LDS into multiple classes based on the causative gene (LDS1-5) providing a general indication of the spectrum of disease severity, from most to least severe form: LDS1=LDS2>LDS3>LDS4>LDS5 [6]. Still, there are not enough data on LDS caused by heterozygous mutations in *SMAD2* to place this form (LDS6) in this spectrum [13]. Recently, it has been suggested that mutations in genes different from *TGFBR1/*2 may give rise to a phenotypic continuum hard to categorize in clear-cut genotype-phenotype correlations [3,6]. In this study, we performed a retrospective multi-center study on clinical and mutational analyses in 24 novel LDS families with 34 patients. Furthermore, a systematic overview of LDS patient’s features for each mutated gene was conducted to verify if observed genotype-phenotype correlations fitted current knowledge. 

Overall, we noticed significant overlaps for *TGFBR1/2*, *SMAD3,* and *TGFB2* patients (Table 1), while emerging genotype-phenotype correlations are described below, subdivided for each gene.

### 4.1. TGFBR1/2 Genes

According to previous studies, most of the identified mutations affected *TGFBR1* (9/24) and *TGFBR2* (10/24) genes. While nearly all mutated patients displayed ascending/aortic root aneurysm, severe arterial involvement was recorded in half. Early aortic dissection was more rarely reported together with arterial tortuosity. Interestingly, we noticed a high number of different arteries being affected. These findings highlight the need for extended arterial imaging, as was already recommended [5]. Valve abnormalities, in particular mitral valve prolapse and insufficiency, affected nearly half of the patients; isolated mitral valve prolapse was also present in seven out of 10 *TGFBR2* patients. As previously described, craniofacial features may be considered to be characteristics of *TGFBR1/2*-LDS including dolichocephaly, hypertelorism, malar hypoplasia and highly arched palate, while abnormal palate/bifid uvula was not as frequently found as described in the literature. Recurrent skeletal anomalies included scoliosis, pes planus, long slender fingers, marfanoid habitus, and pectus deformity. Hernias were of different types (diaphragmatic, inguinal, or umbilical) and together with joint laxity were registered in half of patients. Cutaneous findings appeared extremely frequent including translucent skin, easy bruising and striae; together with facial milia, described in half mutated patients, skin features appeared as extremely useful handle for diagnosis [14]. Overall, our data confirm broad overlap between LDS phenotypes caused by *TGFBR1/2* pathogenic variants, as previously assessed [6]. Also, in four families the presence of significant craniofacial malformations correlated with a higher risk of vascular manifestations [5,6]. Finally, two children had tooth abnormalities, in particular enamel defects, a possibly underestimated features in LDS in line with the role of TGF-β signaling in dental and enamel formation [15].

### 4.2. SMAD3 Gene

Among our three *SMAD3*-mutated families, early osteoarthritis, considered a hallmark of this subset of patients, was described only in one, although it segregated in the proband and his father. In line with our data, despite early studies reported osteoarthritis in all *SMAD3* patients [4], others noticed a lower incidence [16]. Interestingly, this family presented very mild cardiovascular anomalies, with only mitral valve prolapse registered in the proband. Generally, *SMAD3*-LDS patients displayed a less severe degree of cardiovascular complications. In fact, despite all the presence of thoracic aortic (and renal arteries) aneurysms, dissection was reported only once, as well as arterial tortuosity and varices. Additional clinical findings overlapped with those recorded in other LDS patients, including recurrent craniofacial dysmorphism such as hypertelorism, malar hypoplasia, micrognathia and highly arched palate, cleft palate and/or uvula, cutaneous features with translucent skin, striae and facial milia, hernias and skeletal manifestation like pes planus, pectus deformity, marfanoid habitus, and scoliosis. It is worth noting that one subject had spontaneous pneumothorax, recorded in a single *SMAD3* patient in the literature [3]. The causes of spontaneous pneumothorax in LDS have not been clearly elucidated but its association with LDS independently from the genetic subtype as well as the occurrence in other HCTD such as Marfan syndrome argues in favor of a common role for TGF-beta signaling. Several lines of evidence based on mouse models, knockout for genes encoding members of the TGF-beta pathway, support this observation (reviewed in reference [17]). In particular, perturbed TGF-beta signaling was implicated in abnormal pulmonary alveolarization and alveolar destruction, leading with age to emphysema and spontaneous lung rupture (i.e., primary spontaneous pneumothorax).

### 4.3. TGFB2 Gene

We identified only one patient with a *TGFB2* pathogenic variant, whose cardiovascular features consisted in mitral valve prolapse and insufficiency, mild arterial tortuosity and varicose veins, in the absence of aneurysms. Additional features included bifid uvula, striae, translucent and hyperextensible skin, facial milia, hernias, joint hypermobility, pectus deformity, scoliosis, and pes planus observed in other LDS genetic subtypes. In line with previous observations, this patient presented the mildest end of LDS phenotypic spectrum [18]. However, it should be noted that phenotypic variability ranging from mild to severe expression was also recorded in *TGFB2*-LDS [19]. Further broadening the clinical picture of this genetic subtype, she also showed cervical spine instability, which was not previously registered in association with *TGFB2*, although it has been commonly encountered in LDS [3]. Interestingly, in Tgfb2 heterozygous knockout mice severe skeletal defects are also observed including skull base and vertebral malformations, in line with the human phenotype [20]. These data corroborate the importance to investigate and diagnose malformation of cervical spine and/or instability, which should be carefully monitored to prevent severe complications in these patients.

### 4.4. Other Genes

No mutations were identified in the latest described LDS genes, namely *SMAD2* and *TGFB3*. Still, limited numbers are available in literature about genotype-phenotype correlation. To date, 9 (likely) pathogenic variants in *SMAD2* have now been described in 15 subjects displaying a broad range of features, including aneurysms, tortuosity of the entire arterial tree, and coronary artery dissections, even in the absence of prominent connective tissue characteristics [13]. Concerning *TGFB3*, 15 different variants were reported in 56 individuals presenting with phenotypic overlap between LDS and MFS [12]. 

## 5. Conclusions

In this work, we broadened the clinical and molecular spectrum of LDS, corroborated, and expanded previously delineated genotype-phenotype correlations, paving the way for a gene-based classification of different disease subtypes. Larger cohort screenings are needed to unravel the thorough clinical and molecular repertoires in LDS to accurately establish diagnostic criteria, define genotype-phenotype correlations, and collect natural history data for clinical prognostication.

## Figures and Tables

**Table 1:** Comparison of the clinical features observed in previously reported and LDS patients described in this cohort for each distinct gene.

Clinical Features	*TGFBR1*		*TGFBR2*		*SMAD3*		*TGFB2*		*SMAD2*	*TGFB3*
	Lit.	This cohort *n* = 12 (%)	Lit.	This cohort *n* = 12 (%)	Lit.	This cohort *n* = 9 (%)	Lit.	This cohort *n* = 1	Lit.	Lit.
Hypertelorism	++++	10/12 (84)	++++	6/12 (50)	++	4/9 (44)	++	1/1	+	++
Strabismus	+	1/12 (8)	+	1/12 (8)	+	0/9	+	0/1	−	−
Malar hypoplasia	+++	8/12 (67)	+++	9/12 (75)	++	9/9 (100)	++	1/1	++++	++
Bifid uvula/Cleft palate	++++	3/12 (25)	++++	5/12 (42)	++	2/9 (22)	+	1/1	−	++
Dolichocephaly	+++	11/12 (92)	+++	7/12 (58)	+	3/9 (33)	−	0/1	++++	−
Hernia	+++	4/12 (33)	+++	6/12 (50)	++	5/9 (55)	++	1/1	++++	++
Striae	++	5/12 (42)	++	3/12 (25)	++	3/8 (37)	++	1/1	++	+
Pectus deformity	+++	5/12 (42)	+++	7/12 (58)	++	6/9 (66)	++	1/1	++	+++
Scoliosis	+++	10/12 (84)	+++	8/12 (67)	++	3/9 (33)	++	1/1	++	+++
Arachnodactyly	+++	5/12 (42)	+++	6/12 (50)	++	1/9 (11)	++	1/1	++	++
Talipes equinovarus	++	1/12 (8)	++	5/12 (42)	+	1/9 (11)	+	0/1	−	++
Osteoarthritis	++	0/11	++	0/10	++	3/6 (50)	+	0/1	++++	++
Cervical spine malformation/instability	+	1/11 (9)	+	2/9 (22)	+	0/3	−	1/1	−	+
Dural ectasia	++	1/11 (9)	++	3/8 (37)	+++	1/4 (25)	++	1/1	+	−
Mitral valve prolapse or insufficiency	++	5/12 (42)	++	7/10 (70)	++	5/9 (55)	++	1/1	++	++
Arterial tortuosity	++++	3/11 (27)	++++	5/11 (45)	++	1/6 (17)	++	1/1	+	+
Aortic root aneurysm	++++	12/12 (100)	++++	9/12 (75)	+++	7/9 (77)	+++	0/1	++++	++
Arterial aneurysms	+++	5/12 (42)	+++	5/11 (45)	+	2/9 (22)	+	0/1	+	+
Aortic dissection	++++	3/12 (25)	++++	2/12 (17)	++	1/9 (11)	+	0/1	−	++

Lit: Literature. Frequencies of clinical feature associated with LDS were scored as: − absent/infrequent; + <25%; ++ 25–50%; +++ 50–75%; ++++ >75%.

**Table 2:** List of variants identified in this study in each gene and molecular details.

Gender	Age at diagnosis	Family history	Origin	Gene	HGVS	Protein	dbSNP	Patient ID (LOVD)	Variant ID (LOVD)
M	7 years	−	Italy	*TGFBR1*	c.1199A>G	p.(Asp400Gly)	rs121918711	#00245208	#0000498906
F	31 years	+	Italy	*TGFBR1*	c.1120G>A	p.(Gly374Arg) §		#00245211	#0000498909
F	29 years	−	Italy	*TGFBR1*	c.1052A>T	p.(Asp351Val) §		#00245212	#0000498911
M	29 years	+	Italy	*TGFBR1*	c.812G>A	p.(Gly271Asp) §		#00245213	#0000498912
M	47 years	+	Italy	*TGFBR1*	c.705_707del	p.(Ser236del)	rs863223830	#00245343	#0000499180
F	17 years	−	Philippines	*TGFBR1*	c.650G>T	p.(Gly217Val) §		#00245345	#0000499182
M	23 years	−	Italy	*TGFBR1*	c.1057G>C	p.(Gly353Arg) §		#00245346	#0000499183
M	17 years	−	Italy	*TGFBR1*	c.1460G>A	p.(Arg487Gln)	rs113605875	#00245347	#0000499184
M	43 years	+	Italy	*TGFBR1*	c.693_699delinsC	p.(Lys232_Ile233del) §		#00245348	#0000499185
F	51 years	+	Italy	*TGFBR2*	c.1609C>T	p.(Arg537Cys)	rs104893809	#00245350	#0000499187
F	3 years	−	Sri Lanka	*TGFBR2*	c.1582C>T	p.(Arg528Cys)	rs104893810	#00245351	#0000499232
F	3 years	−	Italy	*TGFBR2*	c.1598G>T	p.(Cys533Phe) §		#00245396	#0000499233
M	9 years	−	Italy	*TGFBR2*	c.1336G>T	p.(Asp446Tyr) §		#00245398	#0000499234
F	45 years	+	Italy	*TGFBR2*	c.263+6C>T	r.263_264insguaa * p.(Arg114 *) §	rs758501054	#00245408	#0000499245
F	36 years	+	Italy	*TGFBR2*	c.1564G>A	p.(Asp522Asn)	rs863223854	#00245409	#0000499246
M	1 year	−	Italy	*TGFBR2*	c.1336G>A	p.(Asp446Asn)	rs886039551	#00245410	#0000499247
M	37 years	+	Italy	*TGFBR2*	c.1187G>A	p.(Cys396Tyr) §		#00245411	#0000499248
F	3 years	−	Italy	*TGFBR2*	c.1184T>C	p.(Leu395Pro) §		#00245412	#0000499249
M	12 years	−	Italy	*TGFBR2*	c.1270T>G	p.(Tyr424Asp) §		#00245413	#0000499250
F	31 years	+	Italy	*SMAD3*	c.1247C>T	p.(Ser416Phe)		#00245414	#0000499251
M	13 years	+	Italy	*SMAD3*	c.1009 + 1G>A	r.872_1009del * p.(Arg292_Gly337del) §		#00245415	#0000499252
F	41 years	+	Italy	*SMAD3*	c.803G>A	p.(Arg268His)	rs863223740	#00245416	#0000499253
M	23 years	+	Italy	*SMAD3*	c.862_871+8del	p.(Arg288Glufs*50) §		#00245417	#0000499254
F	48 years	+	Italy	*TGFB2*	c.480del	p.(Phe160Leufs*14) §		#00245418	#0000499255

*: demonstrated by RT-PCR; §: newly reported variants.

## Supplementary Materials

The following are available online at https://www.mdpi.com/2073-4425/10/10/764/s1, Table S1: Primers, Table S2: Clinical and molecular features of LDS patients described in this cohort.
